# Adipokines in multiple sclerosis patients are related to clinical and radiological measures

**DOI:** 10.1007/s00415-022-11519-8

**Published:** 2022-12-23

**Authors:** Floor C. Loonstra, Kim F. Falize, Lodewijk R. J. de Ruiter, Menno M. Schoonheim, Eva M. M. Strijbis, Joep Killestein, Helga E. de Vries, Bernard M. J. Uitdehaag, Merel Rijnsburger

**Affiliations:** 1 MS Center Amsterdam, Neurology Department, Vrije Universiteit Amsterdam, Amsterdam Neuroscience, Amsterdam University Medical Centers (Amsterdam UMC), Location VUmc, De boelelaan 1117, 1081 HV Amsterdam, The Netherlands; 2 MS Center Amsterdam, Molecular Cell Biology and Immunology, Vrije Universiteit Amsterdam, Amsterdam Neuroscience, Amsterdam University Medical Centers, Location VUmc, Amsterdam, The Netherlands; 3 MS Center Amsterdam, Anatomy and Neurosciences, Vrije Universiteit Amsterdam, Amsterdam Neuroscience, Amsterdam University Medical Centers, Location VUmc, Amsterdam, The Netherlands

**Keywords:** Multiple sclerosis, Adipokines, Disability, BMI MRI

## Abstract

**Background:**

An imbalance of adipokines, hormones secreted by white adipose tissue, is suggested to play a role in the immunopathology of multiple sclerosis (MS). In people with MS (PwMS) of the same age, we aimed to determine whether the adipokines adiponectin, leptin, and resistin are associated with MS disease severity. Furthermore, we aimed to investigate whether these adipokines mediate the association between body mass index (BMI) and MS disease severity.

**Methods:**

Adiponectin, resistin, and leptin were determined in serum using ELISA. 288 PwMS and 125 healthy controls (HC) were included from the Project Y cohort, a population-based cross-sectional study of people with MS born in the Netherlands in 1966, and age and sex-matched HC. Adipokine levels and BMI were related to demographic, clinical and disability measures, and MRI-based brain volumes.

**Results:**

Adiponectin levels were 1.2 fold higher in PwMS vs. HC, especially in secondary progressive MS. Furthermore, we found a sex-specific increase in adiponectin levels in primary progressive (PP) male patients compared to male controls. Leptin and resistin levels did not differ between PwMS and HC, however, leptin levels were associated with higher disability (EDSS) and resistin strongly related to brain volumes in progressive patients, especially in several grey matter regions in PPMS. Importantly, correction for BMI did not significantly change the results.

**Conclusion:**

In PwMS of the same age, we found associations between adipokines (adiponectin, leptin, and resistin) and a range of clinical and radiological metrics. These associations were independent of BMI, indicating distinct mechanisms.

## Introduction

Obesity during childhood or adolescence is associated with a ~ 2.5-fold increased risk of developing multiple sclerosis (MS), suggesting that accompanying metabolic and immunological alterations promote disease pathogenesis [1, 2]. Additionally, increased body mass index (BMI) and obesity have been associated with higher disability, increased pro-inflammatory cytokines in relapsing remitting (RR) MS, and reduced gray matter volumes on MRI [3, 4]. However, the underlying mechanisms of these associations are still largely unknown.

Enhanced body weight (BMI > 25) is associated with low-grade inflammation, which is driven by the (altered) release of adipokines in the circulation. Recent data suggest that an imbalance of pro- and anti-inflammatory adipokines, a class of cytokines released by the white adipose tissue (WAT), contributes to immune-pathological processes related to MS and may therefore represents one of the possible links between increased BMI and MS disease severity [5]. Adipokines are key players in the maintenance of energy balance as well as immune homeostasis and especially adiponectin, leptin and resistin have been described to play a pivotal role in both processes [5].

Adiponectin, the most abundant adipokine found in human plasma, exerts anti-inflammatory effects by suppressing the production of pro-inflammatory cytokines. While it suppresses activation of T and B lymphocytes by stimulating secretion of anti-inflammatory IL-10[6, 7], adiponectin may also exert pro-inflammatory effects [8, 9]. Leptin possesses various pro-inflammatory roles in endothelial cells and macrophages by producing pro-inflammatory molecules such as TNF-α, IL-6 and CXCL10 [10]. Moreover, leptin increases type 1 T helper cell activity while it decreases the proliferation of regulatory T cells [10]. While adiponectin and leptin are predominantly produced by the WAT, resistin is believed to be mainly produced in peripheral blood mononuclear cells (PBMCs) and increases when stimulated with pro-inflammatory cytokines. In turn, resistin treatment stimulates the release of other pro-inflammatory cytokines from human PBMC’s [11, 12].

Adipokines may predict progression in other chronic inflammatory diseases[13], but only few reports address the involvement of adipokines in MS. Adipokines interact with the blood–brain barrier (BBB) and may either cross the BBB or affect BBB integrity. Consequently, abnormal adipokine secretion could exert potent CNS effects through oxidative stress and inflammation [14]. Independent studies show that leptin, resistin, and adiponectin levels are increased in people with MS (PwMS). However, results are contrasting and a direct association with MS phenotype is unknown.[15–18] Additionally, studies were confounded by unknown treatment status, sex and most importantly, differences in age [5, 19, 20].

This study, therefore, aimed to investigate the association between adipokines and disease severity in the different MS phenotypes. To remove potential bias effects of age, this was assessed in a cohort of PwMS and healthy controls (HC) of the same age (Project Y) [21]. We explored (1) differences in adiponectin, leptin and resistin levels in MS and how these relate to clinical, disability, and MRI measures, adjusted and unadjusted for BMI and (2) how BMI relates to clinical, disability and MRI measures.

## Methods

### Cohort

Patients were selected from the cohort project Y, a population-based cross sectional birth year cohort aimed to include all PwMS (as defined by the 2017 McDonald Criteria) [22] born in the Netherlands in 1966 and HC’s born in 1965–1967. Details on this cohort have been described previously [21]. All participants with available plasma samples were included for the aim of this study. All participants gave written informed consent and this study was approved by the Medical Ethical Committee of the Amsterdam UMC, location VUmc.

### Adipokine measurements

Serum was collected via standard vena puncture. Adipokine quantification was performed using ELISA. Samples were diluted 1:5000 to 1:10,000 for adiponectin, 1:40 for leptin (Human standard ABTS development kit, PeproTech, London, UK) and 1:20 for resistin (Human standard ABTS development kit, PeproTech) and randomized between plates. The samples of each participant were analyzed in duplo within one run and each plate contained a sample from a control donor, to control for inter-assay variability. Inter-assay and intra-assay variability was 21% and 4.3% for resistin, 29% and 21% for leptin and 12% and 11% for adiponectin, respectively. The personnel performing the analyses was blinded for the clinical data. Leptin levels below limit of detection (78.10 pg/mL) were imputated by assigning random numbers between 0 and 78.10.

### Neuroimaging

228 patients and 113 HC underwent 3 T MR imaging of brain and spinal cord, which is described in detail elsewhere [21]. In short, the MRI protocol included cerebral high resolution anisotropic sagittal three dimensional (3D)-T1 sagittal slices and 3D- Fluid Attenuation Inversion Recovery (3D-FLAIR) sequences. Lesions were automatically segmented on FLAIR for lesion volumes (LV) and filled on T1 images. Normalized total brain volume (NBV), normalized cortical gray matter volume (NCGMV) and normalized white matter volume (NWMV) were calculated using SIENAX, providing normalization for head size. Total normalized deep grey matter (NDGMV) and thalamic volume (NThalV) were calculated using FIRST in FSL6. The Harvard_Oxford atlas was used to measure cerebellar grey matter volume (NCbV) using a previously described procedure [23]. Cerebral 3DT1 images were used to measure mean upper cervical cord area (MUCCA) using SCT, as previously described [24].

### Clinical assessment

A comprehensive interview was conducted and included date of onset, MS phenotype, exacerbations and disease progression, use of disease modifying therapies (DMT), general medical history and use of other medication. For the purpose of this study, we used information on diabetes mellitus (yes/no), hyperlipidemia and statin use. Expanded Disability Status Scale (EDSS) scores were used to assess overall MS-related disability. Upper and lower extremity function was measured using the nine-hole peg test (9HPT) and timed 25 foot walking test (T25FWT), respectively. The T25FWT was completed twice and the 9HPT was performed twice each hand; the average of both trials was used. BMI was obtained by dividing weight in kilograms by length in meters squared.

### Statistical analyses

Statistical analysis was performed using SPSS (Version 26.0, IBM, USA). Histograms and the Shapiro–Wilk test were used to test for normality of distribution. Adiponectin, leptin, resistin and LV were log-transformed to achieve a normal distribution. Independent *T*-tests, ANOVA and non-parametric tests were used to compare characteristics between PwMS and HC and between MS subtypes. A *p* value < 0.05 was considered statistically significant; Bonferroni corrections were applied to adjust for multiple comparisons.

We hypothesized that if adipokines would serve as mediators in the relation between BMI and MS disease severity (1) the analysis would not yield significant relations between adipokines and MS while adjusting for BMI (2) adipokines would be significantly related with BMI (3) both adipokines and BMI would yield similar significant associations with MS disease severity and (4) the relation between adipokines and MS disease severity would significantly change while excluding BMI as covariate. Analyses thus consisted of 4 steps.

#### Step 1: relation between adipokines and clinical and radiological measures

##### Group differences in adipokine levels

General linear models (GLM) were performed to assess differences in adipokine levels between patients and HC, between MS subtypes, EDSS groups, patients using DMT and between RR onset patients with a relapse within 3 months prior to sampling and patients without a relapse. When comparing patients vs. HC, BMI, sex, type 1 or type 2 diabetes mellitus (yes/no), statin use (yes/no) and hyperlipidemia (yes/no) were used as covariates based on their known confounding effects. If comparing patients groups based on clinical parameters, the following covariates were used: BMI, sex, disease duration and DMT use (duration and current DMT yes/no), diabetes mellitus, statin use and hyperlipidemia. No significant changes in adipokine levels were found between smokers and non-smokers and analyses were therefore not corrected for smoking.

##### Univariate regressions

Relations of adipokines with disability (EDSS, 9HPT, T25FWT) and volumetric MRI measures were assessed using univariate linear regression analysis, correcting for BMI, sex, disease duration, DMT use (duration and current DMT yes/no), onset type (RRMS vs. progressive), diabetes mellitus and statin use. Regression analyses were stratified by sex and MS subtype. In each strata, effect modification by sex and onset type was assessed. Cases were classified as outliers if Cook’s distance was ≥ 1.0 and/or residuals were three or more standard deviations from the mean and/or based on visual inspection. Patients unable to perform the T25FWT or 9HPT were excluded from regression analyses.

#### Step 2: relation between BMI and adipokines

##### Correlation analysis

For each sex-specific subtype, Spearman’s correlation was used for correlation analyses between adipokines and BMI.

#### Step 3: relation between BMI and clinical and radiological measures

##### Group differences in MRI volumes

A GLM was used to assess differences in MRI volumes between BMI categories “lean” (BMI < 25), “overweight” (BMI 25–30) and “obese” (BMI ≥ 30). Patients were stratified by sex and adjusted for disease duration, DMT use (duration and current DMT yes/no), diabetes mellitus and statin use.

##### Univariate regressions

Relations of BMI with disability and volumetric MRI measures were analyzed using linear regressions, stratified by sex and MS subtype and adjusted for disease duration, DMT use (duration and current DMT yes/no), diabetes mellitus and statin use wherever appropriate. When stratified by sex, analysis were also corrected for onset type (RRMS vs. progressive) and *vice versa*.

For similar significant associations between adipokines and MRI volumes on the one hand and associations between BMI and MRI volumes on the other hand, we explored whether BMI and adipokines were independently associated with MRI volumes using linear regression analyses including both the respective adipokine and BMI in the same model. Sex, disease duration, DMT use (duration and current DMT yes/no), onset type (RRMS vs. progressive), diabetes mellitus and statin use were also entered as covariates.

Lastly, relations of BMI and adipokines with MRI volumes in HC were assessed using linear regressions.

#### Step 4: relation of adipokines and clinical and radiological measures—unadjusted for BMI

##### Univariate regressions

To assess whether BMI adjustment significantly changed the relation between adipokines and MS disease severity, all analyses of the first step were repeated with similar covariates while excluding BMI.

## Results

### General characteristics

288 PwMS (RRMS: 170; SPMS: 80; PPMS: 37) and 125 HC of the Project Y cohort were included. Table 1 depicts the demographic, clinical and MRI characteristics for all patients and HC.

**Table 1:** General characteristics of people with multiple sclerosis and healthy controls

	Healthy controls (*n* = 125)	All PwMS (*n* = 288)	RRMS (*n* = 170)	SPMS (*n* = 80)	PPMS (*n* = 37)
Age, years (SD)	52.9 ± 1.2	52.9 ± 0.9	52.9 ± 0.9	53.1 ± 0.9	53.1 ± 0.9
Female (%)	92 (74%)	207 (72%)	139 (82%)^†^	48 (60%)	19 (51%)^‡^
BMI (SD)	25.6 ± 3.7	26.1 ± 4.9	26.6 ± 5.2	25.5 ± 4.4	25.4 ± 3.7
Adiponectin (ng/mL), median (IQR)	10,591.7 (8416.6)	12,455.9 (7922.4)*	12,293.4 (7365.7)	13,289.5 (8329.4)	12,681.1 (8071.0)
Leptin (pg/mL), median (IQR)	31,856.7 (50,711.1)	34,987.1 (55,344.0)	42,361.5 (61,425.3)	52,378.2 (52,585.1)	27,216.6 (45,793.4)
Resistin (pg/mL), median (IQR)	4789.2 (2666.0)	4618.9 (2436.4)	4607.5 (2670.4)^†^	4687. (2859.4)	4562.3 (1791.4)^‡^
EDSS, median (IQR)	–	3.75 (2.0)	3.0 (2.0)^†^	6.0 (2.5)	4.0 (2.5)^‡^
Disease duration since symptom onset (IQR)		15.3 (15.9)	14.2 (15.2)^†^	20.7 (11.5)^§^	8.1 (9.0)^‡^
Current DMT, *n* (%)	–	134 (47%)	94 (55%)	33 (41%)	8 (22%)^‡^
First line DMT		90 (31%)	68 (40%)^†^	19 (24%)^§^	2 (5%)^‡^
Second line DMT		46 (16%)	26 (15%)	14 (18%)	6 (16%)
DMT total duration (IQR)	–	6.1 (9.0)	6.4 (9.1)	7.8 (8.8) ^§^	1.5 (2.7)^‡^
Number of relapses, *n* (%)	–				
< 5 relapses		206 (72%)	122 (72%)	46 (58%)^§^	37 (100%)^‡^
> 5 relapses		82 (29%)	48 (28%)	34 (43%)^§^	–^‡^
Statine use, n (%)	1 (1%)	12 (4%)	4 (2%)	6 (8%)	2 (5%)
Diabetes mellitus, n (%)	1 (1%)	8 (3%)	6 (5%)	1 (1%)	1 (3%)

MRI volumes	(*n* = 113)	(*n* = 230)	(*n* = 144)	(*n* = 54)	(*n* = 32)
Normalized brain volume, L (mean, SD)	1.54 ± 0.077	1.48 ± 0.078*	1.49 ± 0.077	1.48 ± 0.067	1.48 ± 0.097
Normalized cortical gray matter volume, L (mean, SD)	0.79 ± 0.051	0.76 ± 0.052*	0.76 ± 0.052	0.74 ± 0.050	0.75 ± 0.055
Normalized deep gray matter volume, mL (mean, SD)	63.53 ± 4.89	59.01 ± 5.43*	59.58 ± 4.86^†^	57.45 ± 6.65	59.03 ± 5.24
Thalamic volume, mL (mean, SD)	21.36 ± 1.67	19.56 ± 2.02*	19.76 ± 1.87	19.01 ± 2.37	19.53 ± 1.93
Normalized white matter volume, L (mean, SD)	0.71 ± 0.043	0.69 ± 0.043*	0.69 ± 0.041	0.70 ± 0.40	0.69 ± 0.055
Normalized cerebellar gray matter volume, mL (mean, SD)	108.40 ± 14.13	101.88 ± 13.64*	102.82 ± 12.43	99.80 ± 16.37	101.21 ± 13.87
Mean upper cervical cord area, mm^2^(mean, SD)	72.9 ± 7.98	67.69 ± 8.64*	69.57 ± 7.84	63.53 ± 9.32	66.24 ± 8.42
Lesion volume, mL (median, IQR)	2.79 (2.43)	10.79 (14.25)*	9.54 (10.43)	14.01 (15.46)	13.70 (18.36)

*BMI* body mass index, *DMT* disease modifying therapy, *EDSS* expanded disability status scale, *SD* standard deviation, *PPMS* primary progressive multiple sclerosis, *RRMS* relapsing remitting multiple sclerosis, *SPMS* secondary progressive multiple sclerosis

*Indicates a significant difference (*p* < 0.05) between all PwMS and healthy controls (uncorrected)

^†^Indicates a significant difference (*p* < 0.05) between RRMS and SPMS (uncorrected)

^‡^Indicates a significant difference (*p* < 0.05) between RRMS and PPMS (uncorrected)

^§^Indicates a significant difference (*p* < 0.05) between SPMS and PPMS (uncorrected)

#### Relation of adipokine levels and clinical and radiological measures: BMI corrected

##### Adipokine levels in PwMS and healthy controls

**Fig. 1:**
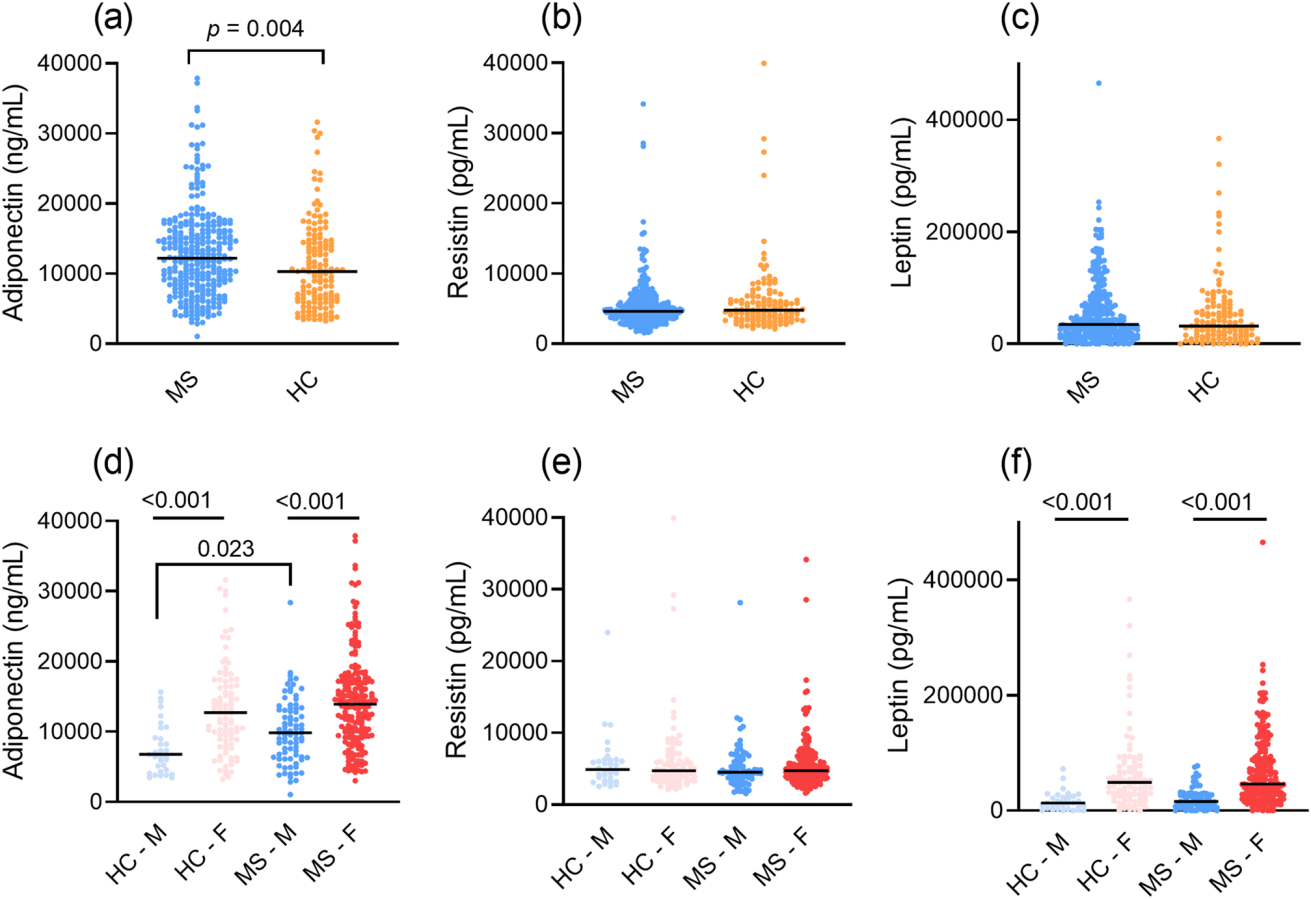
Levels of adiponectin (**a**), resistin (**b**) and leptin (**c**) in healthy controls versus people with multiple sclerosis (PwMS) and levels of adiponectin (**d**), resistin (**e**) and leptin (**f**) in PwMS stratified by sex. Each dot in the scatter box-plot represents a sample. ***p*** values were calculated with a general linear model, adjusted for sex, BMI, diabetes mellitus (yes/no), statin use (yes/no) and hyperlipidemia (yes/no). *F* female, *HC* healthy controls, *M* male, *MS* multiple sclerosis

Adiponectin concentrations were higher in PwMS compared to HC (*p* = 0.004), whereas resistin (*p* = 0.088) and leptin *(p* = 0.945) levels did not differ between PwMS and HC’s (Fig. 1). Male patients had higher adiponectin concentrations compared to male HC (*p* = 0.023), however no differences were found between female PwMS and female HC (*p* = 0.055) (Fig. 1). Both adiponectin and leptin levels were higher in female PwMS compared to male PwMS and in female HC compared to male HC (all *p* < 0.001) (Fig. 1). After BMI stratification, only adiponectin levels were higher in patients with BMI ≥ 25 (*n* = 153) compared to HC with BMI ≥ 25 (*n* = 75) (*p* = 0.002), whereas all adipokine levels in patients with BMI < 25 did not differ from HC’s with BMI < 25.

**Fig. 2:**
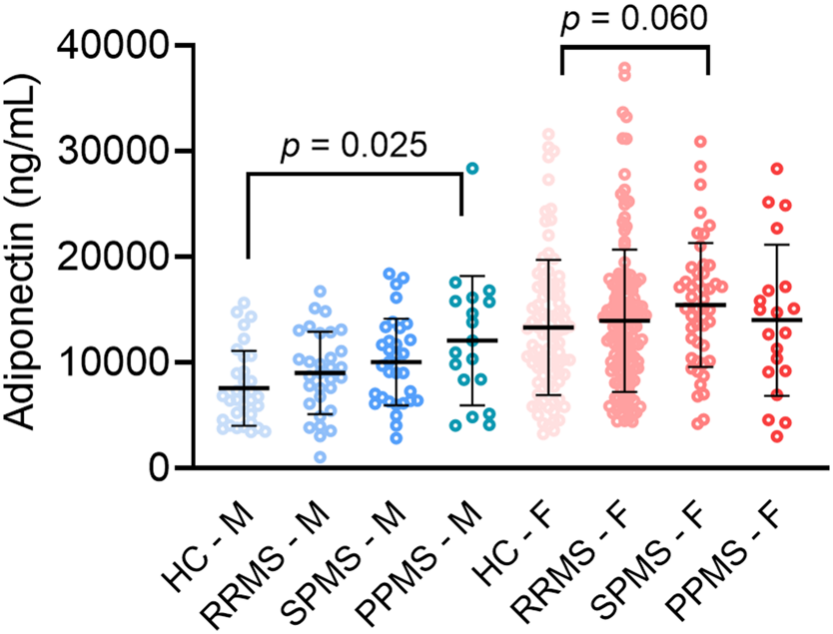
Adiponectin levels in healthy controls and people with multiple sclerosis, stratified by sex and MS subtype. Each dot in the scatter box-plot represents a sample. *p* values were calculated with a general linear model (adjusted for BMI, diabetes status, statin use and hyperlipidemia) followed by post-hoc analyses, Bonferroni corrected. *HC* healthy control, *PPMS* primary progressive multiple sclerosis, *RRMS* relapsing remitting multiple sclerosis, *SPMS* secondary progressive multiple sclerosis

Stratification by MS subtype showed significantly higher adiponectin levels in SPMS compared to HC (*p* = 0.012). When stratified by sex, only in male PPMS higher adiponectin concentrations were observed compared to male HC (*p* = 0.025) and a trend towards increased adiponectin levels in female SPMS compared to HC (*p* = 0.060) (Fig. 2).

##### Adipokine levels across PwMS with disease modifying therapy

Resistin levels were lower in PwMS using teriflunomide (*n* = 15) compared to PwMS using glatiramer acetate (*n* = 22; *p* = 0.020), dimethyl fumarate (*n* = 36; *p* = 0.032) and ocrelizumab (*n* = 14; *p* = 0.042). No differences were detected in adiponectin and leptin concentrations between all DMT’s. Moreover, adipokine levels did not differ between patients without DMT, patients with first-line DMT (interferon-beta, dimethyl fumarate, glatiramer acetate and teriflunomide) and patients with second-line DMT (ocrelizumab, natalizumab, fingolimod).

##### Relation of adipokine levels and clinical measures

Higher adiponectin levels were related to a longer disease duration in female progressive MS (*β* = 0.349, *p* = 0.034), but not in female SPMS or PPMS. Leptin levels inversely correlated with disease duration (β = -0.212, *p* = 0.020) in female SPMS, while resistin did not correlate with disease duration. In addition, none of the adipokines in relapse onset patients with a relapse within 3 months prior to sampling (*n* = 8) did significantly differ from relapse onset patients in remission (*n* = 240).

Regression analyses of clinical disability scores as dependent variables are depicted in Table 2. Higher leptin levels were associated with higher EDSS in all strata, except for SPMS. In female RRMS, increased leptin levels were associated with higher EDSS (*β* = 0.260, *p* = 0.011), but not in male RRMS.

**Table 2:** Association between BMI and disability measures (EDSS, 9HPT and T25FWT)—adjusted univariate linear regression analysis

	EDSS Std. *β*	9HPT Std. *β*	T25FWT Std. *β*
BMI			
Male patients	− 0.101	0.097	0.077
Female patients	− 0.024	− 0.016	0.107
RRMS	− 0.009	0.108	0.112
SPMS	− 0.006	0.015	0.237
PPMS	0.226	− 0.012	− 0.066
Adiponectin			
Male patients	0.070	− 0.003	0.203
Female patients	0.033	− 0.017	0.039
RRMS	− 0.108	− 0.064	− 0.072
SPMS	0.108	0.032	0.187
PPMS	0.001	0.037	0.038
Resistin			
Male patients	0.127	0.337	0.106
Female patients	− 0.128	− 0.106	− 0.125
RRMS	− 0.059	− 0.072	− 0.073
SPMS	0.071	0.031	0.023
PPMS	− 0.015	− 0.205	− 0.195
Leptin			
Male patients	0.259*	0.156	0.030
Female patients	0.171*	0.042	0.005
RRMS	0.269**	0.048	0.049
SPMS	0.100	0.099	− 0.243
PPMS	0.436*	0.206	0.280

Bold values denote statistical significance at the *p* < 0.05 level

The following disease specific and disease modifying factors were included in the univariate linear regression analysis as covariates: disease duration, DMT duration, DMT use (yes/no), BMI, sex, onset type, statin use (yes/no) and diabetes mellitus (yes/no).

*BMI* body mass index, *EDSS* expanded disability status scale, *9HPT* Nine hole peg test, *25FWT* 25 foot timed walking test, *Std. β* standardized beta, *PPMS* primary progressive multiple sclerosis, *RRMS* relapsing remitting multiple sclerosis

*p value < 0.05; **p value < 0.01

##### Relation of adipokine levels and radiological measures

**Fig. 3:**
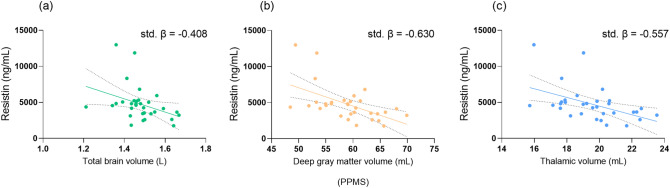
Scatterplot of resistin levels and total brain volume (**a**) deep gray matter volume (**b**) and thalamic volume (**c**) in primary progressive multiple sclerosis. Dashed lines indicate the 95% confidence intervals for the regression line. *PPMS* primary progressive multiple sclerosis

Adiponectin was significantly related to NBV (*β* = − 0.316, *p* = 0.026) and NWMV (*β* = − 0.533, *p* = 0.002) in SPMS (Table 3). Lower leptin levels were associated with lower NCGMV in all female patients. Additionally, in RRMS males, leptin was significantly related to LV (*β* = 0.677, *p* = 0.039). Resistin levels were negatively associated with NBV, NCGMV, NDGMV and NThalV in PPMS (Fig. 3). In RRMS males, resistin was also inversely related to NDGMV (*β* = − 0.637, *p* = 0.028). However in SPMS, lower resistin concentrations were associated with lower thalamic volume (*β* = 0.268, *p* = 0.044).

**Table 3:** Association between adipokines and MRI volumes: adjusted univariate linear regression analysis

	NBV Std. *β*	NWM Std. *β*	NCGMV Std. *β*	NDGMV Std. *β*	NThalV Std. *β*	NCbV Std. *β*	MUCCA Std. *β*	LV Std. *β*
Adiponectin								
Male patients	− 0.123	− 0.074	− 0.128	− 0.087	− 0.021	− 0.013	0.121	0.258
Female patients	0.029	0.033	0.010	0.066	0.029	− 0.003	− 0.039	− 0.066
RRMS	0.475	0.119	− 0.006	0.097	0.049	0.008	− 0.008	0.012
SPMS	− 0.361*	− 0.533**	− 0.103	− 0.239	− 0.229	− 0.087	0.227	0.019
PPMS	− 0.093	− 0.078	− 0.094	0.047	0.107	− 0.031	− 0.236	0.150
Resistin								
Male patients	− 0.052	− 0.079	− 0.001	− 0.174	− 0.026	0.095	0.129	− 0.042
Female patients	− 0.025	− 0.153	0.086	− 0.009	− 0.013	0.002	− 0.080	0.029
RRMS	0.020	− 0.160	0.145	− 0.007	0.008	0.066	− 0.072	− 0.006
SPMS	0.169	0.264	0.081	0.176	0.268*	0.097	− 0.109	− 0.107
PPMS	− 0.408*	− 0.303	− 0.366*	− 0.630***	− 0.557**	− 0.330	0.118	0.295
Leptin								
Male patients	− 0.177	− 0.268	− 0.047	− 0.080	− 0.041	− 0.193	− 0.122	− 0.085
Female patients	0.145	− 0.055	0.272**	0.067	− 0.001	0.118	− 0.018	0.018
RRMS	0.078	− 0.102	0.195	− 0.044	− 0.090	0.089	0.005	0.008
SPMS	0.302	0.085	0.285	0.325	0.264	0.146	0.187	− 0.107
PPMS	− 0.029	− 0.169	0.135	− 0.102	− 0.139	− 0.039	− 0.381	0.206

Bold values denote statistical significance at the *p* < 0.05 level

The following disease specific and disease modifying factors were included in the univariate linear regression analysis as covariates: disease duration, DMT duration, DMT use (yes/no), BMI, sex, onset type, statin use (yes/no) and diabetes mellitus (yes/no)

*Std. β* standardized Beta, *PPMS* primary progressive multiple sclerosis, *RRMS* relapsing remitting multiple sclerosis, *SPMS* secondary progressive MS, *LV* Lesion volume, *MUCCA* Mean upper cervical cord area, *NBV* normalized total brain volume, *NCbV* cerebellar gray matter volume, *NCGMV* normalized cortical gray matter volume, *NDGMV* normalized deep gray matter volume, *NThalV* normalized thalamic volume, *NWMV* normalized white matter volume

*p value < 0.05; **p value < 0.01; ***p value < 0.001

### Relation of BMI with adipokines

First, univariate analyses were performed to assess the relation between adipokine levels and BMI (Table 4). Adiponectin negatively correlated with BMI, whereas resistin and leptin correlated positively.

**Table 4:** Correlations between adipokines and BMI in different subgroups

	HC		MS all		RRMS		SPMS		PPMS	
	Male	Female	Male	Female	Male	Female	Male	Female	Male	Female
Adiponectin	− 0.29	− 0.26*	− 0.10	− 0.28***	0.10	− 0.29***	− 0.05	− 0.31*	− 0.34	− 0.11
Leptin	0.74***	0.58***	0.45***	0.66***	0.31	0.67***	0.59***	0.57***	0.46	0.66***
Resistin	0.12	0.21*	− 0.01	0.26***	0.08	0.31***	− 0.18	0.26	0.14	− 0.44

Bold values denote statistical significance at the *p* < 0.05 level

*BMI* body mass index, *HC* healthy controls, *PPMS* primary progressive multiple sclerosis, *RRMS* relapsing remitting multiple sclerosis, *SPMS* secondary progressive multiple sclerosis

Values are Spearman rho, *p value < 0.05; **p value < 0.01; ***p value < 0.001

### Relation of BMI with clinical and radiological measures

In female PPMS, increased BMI was significantly associated with higher EDSS (*β* = 0.464, *p* = 0.032). No other relations between BMI and clinical disability measures were observed (Table 2). Next, MRI volumes were compared between BMI categories. Obese female patients (BMI ≥ 30, *n* = 28) had significantly lower NCGMV compared to female patients in the lowest BMI group (BMI < 25, *n* = 76) (*p* = 0.023). In addition, overweight male patients (BMI 25–30, *n* = 28) had significantly lower NCGMV compared to lean male patients (BMI < 25, *n* = 27) (*p* = 0.030).

Regression analyses of volumetric MRI measures are shown in Table 5. BMI was positively associated with NWMV in female and in PPMS, while BMI was inversely related to NCGMV in SPMS. Additional analysis revealed that in female SPMS, higher BMI was associated with higher LV (*β* = 0.395, *p* = 0.040), whereas increased BMI was associated with higher NWMV (*β* = 0.619, *p* = 0.030) in female PPMS.

**Table 5:** Association between BMI and disability measures and MRI volumes—adjusted univariate linear regression analysis

	EDSS Std. *β*	9HPT Std. *β*	T25FWT Std. *β*	NBV Std. *β*	NWMV Std. *β*	NCGMV Std. *β*	NDGMV Std. *β*	NThalV Std. *β*	NCbV Std. *β*	MUCCA Std. *β*	LV Std. *β*
BMI											
Male patients	− 0.101	0.097	0.077	− 0.027	0.184	− 0.179	− 0.047	0.087	− 0.174	0.106	− 0.116
Female patients	− 0.024	− 0.016	0.107	0.008	0.168*	− 0.140	0.061	0.137	− 0.115	− 0.014	− 0.024
RRMS	− 0.009	0.108	0.112	− 0.012	0.118	− 0.125	0.078	0.118	− 0.100	− 0.042	− 0.046
SPMS	− 0.006	0.015	0.237	− 0.111	0.205	− 0.298*	− 0.050	0.027	− 0.239	0.038	0.279
PPMS	0.226	− 0.012	− 0.066	0.309	0.407*	0.134	0.172	0.526**	0.034	0.167	− 0.358

Bold values denote statistical significance at the *p* < 0.05 level

The following disease specific and disease modifying factors were included in the univariate linear regression analysis as covariates: disease duration, DMT duration, DMT use (yes/no), BMI, sex, onset type, statine use (yes/no) and diabetes mellitus (yes/no)

*EDSS* Expanded disability status scale, *9HPT* Nine hole peg test, *25FWT* 25 Foot timed walking test, *Std. β* standardized Beta, *PPMS* primary progressive multiple sclerosis, *RRMS* relapsing remitting multiple sclerosis, *SPMS secondary progressive multiple sclerosis* *LV* Lesion volume, *MUCCA* Mean upper cervical cord area, *NBV* normalized total brain volume, *NCbV* Cerebellar gray matter volume, *NCGMV* Normalized cortical gray matter volume, *NDGMV* Normalized deep gray matter volume, *NThalV* Normalized thalamic volume, *NWMV* Normalized white matter volume

*p value < 0.05; **p value < 0.01

The only corresponding significant association between BMI and MRI volumes and adipokines and MRI volumes was the association with NThalV in PPMS. Both BMI (*β* = 0.630, *p* = 0.001) and resistin (*β* = − 0.515, *p* = 0.002) were significantly associated with NThalV when analyzed in the same association model, indicating that BMI and resistin are independently associated with NThalV in PPMS.

#### Relation of BMI and adipokines with MRI volumes in healthy controls

In HCs, higher BMI was associated with higher NWMV (β = 0.230, *p* = 0.016), whereas higher BMI was related to lower CGMV (*β* = − 0.197, *p* = 0.026) and lower NCbV (*β* = − 0.289, *p* = 0.001). In male HC, higher BMI was associated with higher LV (*β* = 0.382, *p* = 0.037).

Finally, adipokine levels were not associated with MRI volumes in HC, although in female HC, leptin levels showed a trend towards significant negative association with MUCCA (*β* = − 0.221, *p* = 0.054).

### Relation of adipokines with clinical and radiological measures: BMI uncorrected

All analyses (step 1) were repeated without BMI as covariate. Overall, without BMI correction, relations did not significantly change. The association between leptin and EDSS in male and female patients even lost its significance without adjusting for BMI and the association between leptin and EDSS in RRMS became weaker. In addition, the relation between adiponectin and NBV in SPMS and the relation between resistin and NthalV in PPMS lost its significance without BMI correction. The only association that became significant without BMI correction was the relation between resistin and NWMV in SPMS (Uncorrected: *β* = 0.288, *p* = 0.049; corrected: *β* = 0.264, *p* = 0.074), nevertheless, no significant association was reported between BMI and NWMV in SPMS.

## Discussion

In a nation-wide MS cohort including patients and HC of the same age, we demonstrate independent associations of adipokines (adiponectin, resistin and leptin) and BMI with disability measures and MRI volumes. Although only adiponectin differed between PwMS and controls, adipokine levels showed several relations with clinical and radiological measures in specific subgroups of patients, with an opposite effect of leptin and resistin compared to adiponectin. These relations were observed while correcting for BMI, suggesting an additional role for adipokines in MS. Importantly, adipokine levels were only related to MRI volumes in PwMS and not in HC, further underlining the possible specific role of adipokines in MS.

### Adiponectin levels

We found increased adiponectin levels in SPMS and male PPMS compared to controls. While several studies described reduced[15, 25, 26] or unaltered levels[27] in MS, the majority of studies reported increased adiponectin levels in patients in remission [28–32]. Earlier studies found positive associations of adiponectin with progression and disease severity in MS, as well as with inflammation and progression in rheumatoid arthritis, chronic kidney disease and inflammatory bowel disease [31, 33, 34]. The positive correlation with disease duration in female progressive MS and negative correlations with brain volumes in our cohort adds to these earlier findings. The presence of high adiponectin levels in MS might be indicative of an attempt, albeit ineffective, of the body to respond to (chronic) inflammation. Of note, our data mainly shows an association of adiponectin with progressive MS in which the inflammatory component is less pronounced. The observed sex-specific associations in progressive patients might be due to different cellular responses to adipokines between the sexes, since accumulating evidence points towards differential function and morphology of cell types explained by sex [35].

Our data may also indicate a dual role of adiponectin in pathological conditions. Since adiponectin is thought to primarily exert an anti-inflammatory effect, the increase of adiponectin in PwMS seems paradoxical. It has, however, been shown that adiponectin exhibits both anti-inflammatory and pro-inflammatory effects dependent on cell type and adiponectin receptor (Adipo-R) expression [36, 37]. Research has demonstrated that expression of Adipo-R1 and 2 is induced in pro-inflammatory mouse macrophages and adiponectin increased TNFα, IL-6 and IL-12, while in anti-inflammatory macrophages, Adipo-R expression is preserved and adiponectin induced the anti-inflammatory IL-10 [9]. Another study showed that adiponectin deficiency in mice led to exacerbated inflammatory responses in microglia in vivo while adiponectin treatment counteracted inflammatory cytokines in microglia, but worsened the response in astrocytes in vitro [38]. Thus, the actions of adiponectin are highly dependent on cell type and phenotype-specific receptor expression.

### Leptin levels

Contrary to previous research, we found no apparent differences in leptin levels between PwMS and controls [39–41]. As most studies did not correct for confounders, the question remains whether previous reported higher leptin levels are actual differences or result from these confounders. Nevertheless, the relatively high age of our cohort and associated decrease in inflammation could have contributed to these discrepancies. Except for SPMS, we did find positive correlations between leptin and EDSS, as well as with LV in male RRMS. We also found a positive correlation with NCGMV in female patients, in line with earlier observations [42, 43]. While these results warrant further exploration, it is possible that leptin exerts differential functions during the more inflammatory disease phase compared to the progressive phase as well as in white versus grey matter. Several animal studies have shown that leptin-deficient mice carrying the obese mutation (*ob/ob*) are not susceptible for EAE, whereas subsequent intraperitoneal leptin replacement induced clinical symptoms. In contrast, intracerebral leptin injections stimulated proliferation of neuronal precursors [44] and reduced infarct volume in ischemic mice [45]. Such neuroprotective effects are further reinforced by studies which found that leptin is associated with larger brain volumes in healthy individuals [46] and regional GM volumes in elderly subjects [47].

It is well-established that adipokine levels significantly differ between sexes [20]. For instance, leptin and adiponectin levels are increased in females compared to males, which is again confirmed in our cohort. This sexual dimorphism is not entirely explained by either sex hormones or body fat distribution and may involve the additional release of leptin from non-adipose sources such as the brain [48].

### Resistin levels

To our knowledge, previous reports on correlations between resistin and MRI volumes are lacking. We found negative associations between resistin and grey matter volumes in PPMS. The role of resistin in neurodegeneration has not been elucidated yet and several modes of action could contribute to this pathophysiological process. In macrophages, resistin induces inflammatory cytokines and increases the expression of cell adhesion molecules [49]. Moreover, resistin is shown to induce endothelial dysfunction in blood vessels and promotes endothelial-monocyte adhesion and infiltration [49, 50]. Importantly, resistin leads to mitochondrial dysfunction, which contributes to progressive neurodegeneration [49, 51]. Thus, resistin could contribute to neurodegeneration via BBB dysfunction and subsequent immune cell infiltration and mitochondrial dysfunction [14]. In contrast, we observed positive associations between resistin and NBV and NThalV in SPMS. Larger SPMS and PPMS groups are required to explore whether resistin has differential effects in progressive phenotypes.

### Disease modifying therapy

An important potential confounder in numerous studies is a lack of controlling for treatment status, which can significantly affect the levels of adipokines, since many treatments are based on immunosuppression [52]. Nevertheless, in our cohort, adipokine levels did not significantly differ between patients without DMT, patients with first-line DMT and patients with second-line DMT. Interestingly, we observed a significant reduction in resistin levels in patients treated with teriflunomide. Teriflunomide is hypothesized to ameliorate MS by reducing proliferation of activated lymphocytes [53], but also exerts direct inhibitory effects on pro-inflammatory cytokine release in monocytes [54]. As main sources of resistin are monocytes and macrophages, the decrease of resistin specifically in this treatment group might be explained by the anti-inflammatory effect of teriflunomide on monocytes.

### BMI

Our findings that (1) adipokines are associated with clinical-, disability- and MRI measures while corrected for BMI (2) BMI associates with different outcome measures compared to adipokines and (3) associations between adipokines and MS metrics do not significantly change without BMI as covariate, suggesting that other (pathophysiological) mechanisms in MS, independent of BMI, are responsible for adipokine alterations. Our initial hypothesis implied that increased BMI in MS may lead to altered adipokine release, which results in the activation of inflammatory pathways [5]. Higher levels of pro-inflammatory cytokines in MS further enhance pro-inflammatory adipokine secretion, creating a positive feedback loop [5]. This would explain the absence of a direct link between BMI, adipokines and MS disease severity in our cohort. However, it remains unclear which stimulus induces this proposed positive feedback loop.

In both HC and in PwMS, BMI was positively associated with NWMV and negatively associated with NCGMV. The positive relation between BMI and NWMV seems paradoxical and results should interpreted with caution. However, other studies have described similar positive relations, which hypothesized that pathological lipid metabolism in the brain of obese individuals may result in increased NWMV [55, 56]. Associations between higher BMI and reductions in normalized GM volume in MS as well as reduction in NBV in the healthy population have been previously described [4, 57]. The specific mechanisms through which obesity affects brain atrophy remain however poorly understood. The lack of association between adipokine levels and MRI volumes in HC suggest that adipokine alterations do not provide a direct link between increased BMI and brain atrophy in healthy individuals.

### Strengths and limitations

The main strength of this study is that all patients and HC are of the same age. Age has a well-known effect on brain volume and on the immune system [58, 59]. Importantly, age significantly affects synthesis and function of adipokines; nearly all adipokines are increased in the older population compared to younger individuals with similar fat mass [19]. However, our study has certain drawbacks. Results are based on cross-sectional data and we could therefore not discriminate cause and effect. Moreover, while our cohort is one of the largest cohorts assessing adipokine levels to date, stratification may have led to loss of statistical power. Lastly, results cannot be generalized to younger MS populations that have generally a more active inflammatory profile.

## Conclusion

In a cohort of PwMS and HC of the same age, we demonstrated associations of adipokines with clinical measures and brain volumes, indicating that adipokines are involved in MS. Associations between adipokines with a range of clinical and radiological metrics were independent from BMI, suggesting a different mechanism in the relation with MS disease severity. Our results aid to the understanding of the neuroprotective and neurotoxic effects of adipokines on the MS brain and could stimulate the development of targeted therapies based on hormonal interventions.

## Data Availability

Anonymized data supporting the findings of this study are available from the corresponding author for the purpose of research only, upon reasonable request.
